# Circulating CO3-610, a degradation product of collagen III, closely reflects liver collagen and portal pressure in rats with fibrosis

**DOI:** 10.1186/1755-1536-4-19

**Published:** 2011-08-03

**Authors:** Toni Segovia-Silvestre, Vedrana Reichenbach, Guillermo Fernández-Varo, Efstathios Vassiliadis, Natasha Barascuk, Manuel Morales-Ruiz, Morten A Karsdal, Wladimiro Jiménez

**Affiliations:** 1 Nordic Bioscience A/S, Herlev, Denmark; 2 Biochemistry and Molecular Genetics, Hospital Clinic, IDIBAPS, CIBERehd, Barcelona, Spain; 3 Department of Physiological Sciences I, Medical School, University of Barcelona, Barcelona, Spain

## Abstract

**Background:**

Hepatic fibrosis is characterized by intense tissue remodeling, mainly driven by matrix metalloproteinases. We previously identified CO3-610, a type III collagen neoepitope generated by matrix metalloproteinase (MMP)-9, and tested its performance as a fibrosis marker in rats with bile-duct ligation. In this study, we assessed whether CO3-610 could be used as a surrogate biomarker of liver fibrosis and portal hypertension in carbon tetrachloride-induced experimental fibrosis.

**Results:**

For this study, 68 Wistar rats were used. Serum CO3-610 was measured by ELISA. Liver fibrosis was quantified by Sirius red staining. Serum hyaluronic acid (HA) was measured with a binding-protein assay. Gene expression of collagens I and III, Mmp2 and Mmp9, and tissue inhibitors of matrix metalloproteinase 1 (Timp1) and 2(Timp2) was quantified by PCR. Hemodynamic measurements were taken in a subgroup of animals. A close direct relationship was found between serum CO3-610 and hepatic collagen content (*r* = 0.78; *P* < 0.001), superior to that found for serum HA (*r* = 0.49; *P* < 0.05). CO3-610 levels in rats with severe fibrosis (43.5 ± 3.3 ng/mL, *P* < 0.001) and cirrhosis (60.6 ± 4.3 ng/mL, *P* < 0.001) were significantly higher than those in control animals (26.6 ± 1.3 ng/mL). Importantly, a highly significant relationship was found between serum CO3-610 and portal hypertension (*r* = 0.84; *P* < 0.001). Liver Mmp9 expression increased significantly in fibrotic animals but decreased to control levels in cirrhotic ones.

**Conclusions:**

Circulating CO3-610 behaves as a reliable indicator of hepatic remodeling and portal hypertension in experimental fibrosis. This peptide could ultimately be a useful marker for the management of liver disease in patients.

## Background

Identification of non-invasive biochemical markers of liver fibrosis is a major challenge for scientists and clinicians dealing with hepatic diseases. The degree of liver fibrosis has emerged as the primary determinant in the diagnosis, prognosis and management of patients with chronic liver diseases [1,2]. Furthermore, the probably availability of antifibrotic treatments in the near future 3 emphasizes the necessity of having accurate tools that allow the sequential evaluation of changes in collagen turnover induced by these drugs.

Liver biopsy (LB) is the gold standard method for the diagnosis of liver fibrosis, providing a unique source of information on fibrosis and assessment of histology. Even for patients in whom serologic tests point to a specific liver disease, LB can give valuable additional information regarding staging, prognosis and management [4]. However, LB can be painful and carries a risk, albeit slight, of complications, some of which may be life-threatening, and therefore it is often reserved for cases in which clinical cirrhosis or the diagnosis of the type of liver disease is unclear. The size and quality of the biopsy, interpretation of histology, and the heterogeneity of fibrosis deposition between patients are variables that can lead to errors and inaccurate assessment of fibrosis [5]. In the past few years, the list of non-invasive surrogate markers of liver fibrosis has increased progressively. Indirect biomarkers, such as platelet count or liver transaminases, reflect alterations in hepatic function but not changes in extracellular matrix (ECM) turnover [6,7]. By contrast, direct biomarkers are thought to be involved in the deposition or removal of the ECM. The extensive deposition of fibrous tissue, together with the active remodeling and recurrent scarring that develops during fibrosis, results in increased serum levels of the constituents and degradation products of fibrous tissue [7,8]. This group includes collagens, proteoglycans, matrix glycoproteins, HA, matrix metalloproteinases (MMPs) and their inhibitors (tissue inhibitors of matrix metalloproteinases; TIMPs), and several cytokines involved in fibrosis signaling pathways. The most extensively studied collagen-derived markers in liver fibrosis are the N-terminal propeptide of type III procollagen (PIIINP) and type IV collagen [8-11]. Indirect and direct markers of liver fibrosis have been validated alone or in combination in different groups of patients with differing degrees of hepatic dysfunction. However, wide agreement on the most suitable biomarker of liver fibrosis is far from being reached.

Recently, a new strategy based on the identification of neoepitopes arising from the cleavage of ECM components by matrix proteolytic enzymes has been proposed [12]. This strategy assumes that collagen cleavage occurs at significant levels only during the gross alterations in ECM homeostasis seen during fibrosis. Neoepitopes, which are cleared into the circulation in peptide fragments, are almost completely absent under healthy conditions, and detectable only when active fibrosis is ongoing. This, in turn, raises the possibility of discriminating between different degrees of disease activity. Based on this approach, we recently identified CO3-610, a specific peptide fragment of type III collagen generated by MMP-9, as a potential liver fibrosis biomarker, and developed an ELISA assay to quantify it in serum [13]. Initial tests of this assay, particularly its performance in rats with bile-duct ligation (BDL) have been previously reported [14].

In the present study we assessed whether the circulating levels of CO3-610 could be used as a surrogate marker of liver fibrosis and portal hypertension in experimental fibrosis induced by carbon tetrachloride (CCl_4_) in rats.

## Results

### Fibrosis quantification and staging

**Figure 1:**
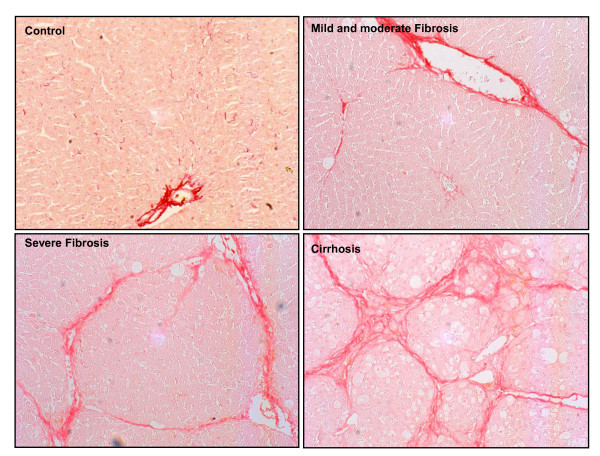
**Classification of the study animals based on liver-collagen content**. Photomicrographs of liver sections stained with Sirius red are representative of control and treatment groups. Rats under CCl_4_ inhalation treatment were classified based on the evidence into three different groups according to the histological quantification of their liver-collagen content: mild/moderate fibrosis (< 6% fibrotic area), severe fibrosis (6 to 11% fibrotic area) and cirrhosis (> 11% fibrotic area). Original magnification ×100.

The liver of animals treated with CCl_4_ had a finely granulated surface macroscopically. As anticipated, the individual response to the fibrosis-induction protocol varied widely from animal to animal. Hence, based on histological analysis, CCl_4_-treated rats were staged into three groups according to the percentage of fibrotic area compared with the total area of the LB: mild and moderate fibrosis (defined as the fibrotic area being < 6% of the total; n = 33), severe fibrosis (6 to 11%; n = 15) and cirrhosis (> 11%; n = 20). Control rats displayed no appreciable alterations in liver histology, and had an almost negligible amount of fibrous tissue. However, in fibrotic rats, there was progressive accumulation of ECM as a consequence of the continuous liver injury, evolving from a slight deposition of fibrosis mainly in the portal area (mild/moderate fibrosis) to numerous and thicker septa resulting from the longer exposure to CCl_4_ (severe fibrosis). Finally, most of the animals exposed to the toxin for the longest periods developed cirrhosis, characterized by the formation of regenerative nodules of liver parenchyma separated by fibrotic septa. Figure 1 shows representative Sirius red staining of tissue from a control rat, a rat with mild/moderate fibrosis, a rat with severe fibrosis and a rat with cirrhosis.

The fibrotic/cirrhotic rats included in the study had important alterations in liver-function tests, being more pronounced in the group of rats with cirrhosis (Table 1). These animals also had a moderate but significant alteration in extracellular fluid homeostasis, as reflected by hyponatremia.

**Table 1:** Body weight, liver-function test results and serum electrolyte values in the rats of the study

	Control (n = 20)	Fibrosis		
		
		Mild/moderate (n = 33)	Severe (n = 15)	Cirrhosis (n = 20)
**Body weight, g**	399 ± 8	369 ± 6	367 ± 9	383 ± 9
**ALT, U/L** a	12.8 ± 1	88.6 ± 31.8	138.1 ± 71*	119.6 ± 31.5***
**AST, U/L** b	117 ± 23	219 ± 27	355 ± 92**	512 ± 69***
**LDH, U/L **c	1093 ± 106	1455 ± 154*	1386 ± 134*	917 ± 112
**Albumin, g/l**	36.1 ± 0.5	34.9 ± 0.5	33.3 ± 1.0	29.0 ± 0.8***
**Serum Na^+^, mEq/l**	142 ± 1.6	142.6 ± 0.5	142.9 ± 0.4	141.5 ± 0.5*
**Serum K^+^, mEq/l**	5.7 ± 0.2	6.0 ± 0.1	5.7 ± 0.1	4.8 ± 0.2
**Serum osmolality, mOsm/Kg**	292 ± 6	294 ± 3	291 ± 3	289 ± 1

^a ^Alanine aminotransferase.

^b^Aspartate aminotransferase.

^c^Lactate dehydrogenase.

*P < 0.05; **P < 0.01 and ***P < 0.001 compared with control rats (one-way ANOVA with Newman-Keuls *post hoc* test and Kruskal--Wallis test with Dunn *post hoc* tests as appropriate).

### CO3-610 is a 25 kDa circulating fragment of collagen type III detected in the serum of cirrhotic rats

**Figure 2:**
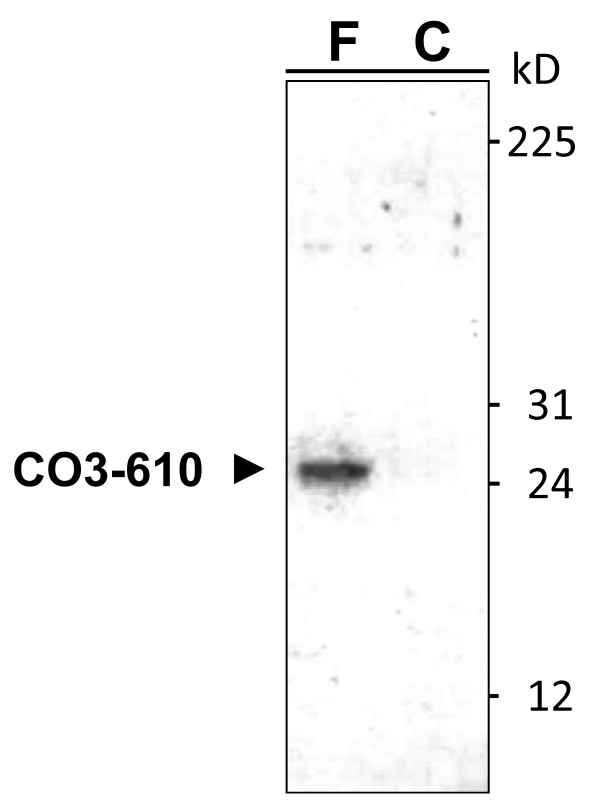
**Immunoprecipitation of CO3-610 type III collagen fragment from rat serum**. CO3-610 was precipitated with monoclonal antibodies from serum of a cirrhotic (F) rat treated with CCl_4_ for 19 weeks and an untreated age-matched control (C) rat and detected by western blotting as a single discrete protein band of 25 kDa. The numbers on the right are the molecular masses of Full-Range Rainbow Markers (Mr 12,000 to 225,000) from GE Healthcare (Little Chalfont, United Kingdom). The figure is representative of three independent experiments.

Western blotting analysis of serum samples from cirrhotic and control rats precipitated with the anti-CO3-610 antibody was carried out (Figure 2). No signal was detected in healthy animals, but a clear specific band at a molecular mass of approximately 25 kDa was visible in blots of serum from cirrhotic rats. These results indicate that a single molecular entity is detected with the CO3-610 antibody, in contrast to other serum collagen ELISA assays reported previously, in which several fragments of collagen were measured simultaneously [15].

### Serum CO3-610 values rose in parallel with liver fibrosis

**Figure 3:**
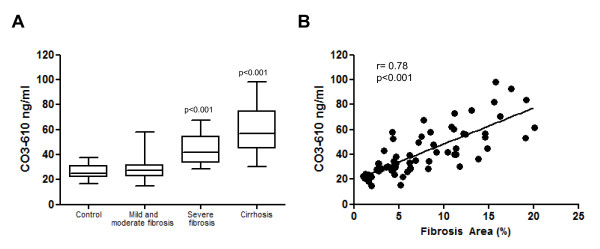
**CO3-610 serum levels in experimental fibrosis induced by chronic administration of carbon tetrachloride in rats**. **(A)** Serum concentrations of CO3-610 in control (untreated) rats (*n* = 20), and rates with mild/moderate fibrosis (*n* = 33), severe fibrosis (n = 15) and cirrhotic (*n* = 20). The box contains the values between the 25th and 75th percentiles, and the horizontal line is the median. Whiskers show the range of the data. Significance is denoted versus the control animals. **(B)** Correlation of CO3-610 serum levels with histological quantification of liver fibrosis in all CCl_4_-treated rats.

No difference was found between serum levels of CO3-610 between control rats and those with mild/moderate fibrosis (26.6 ± 1.3 ng/ml versus 28.5 ± 1.6 ng/ml, respectively) (Figure 3A). However, progression of liver fibrosis was associated with a parallel increase in the circulating levels of the collagen III-derived epitope; values significantly increased in rats with severe fibrosis (43.5 ± 3.3 ng/ml, *P* < 0.001) and reached maximum levels in rats with cirrhosis (60.6 ± 4.3 ng/ml, *P* < 00.01). There was a close direct relationship between CO3-610 values and hepatic collagen content in CCl_4_-treated rats (*r* = 0.78; *P* < 0.001) (Figure 3B). These differences were clearly greater than those obtained by calculating the correlation coefficient between serum hyaluronic acid (HA) (a well-established serum marker of liver fibrosis) and the hepatic collagen content in fibrotic/cirrhotic rats (*r* = 0.49; *P* < 0.05).

### Serum CO3-610 values closely correlate with portal hypertension

**Figure 4:**
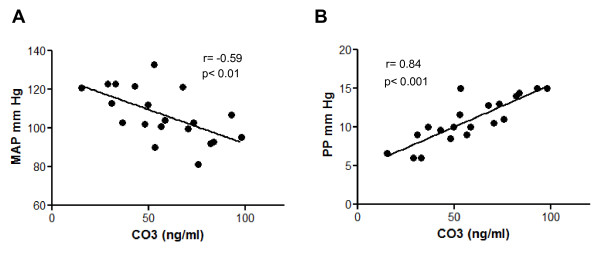
**Correlation of CO3-610 serum levels with portal pressure (PP) and mean arterial pressure (MAP) in a group of 20 CCl_4_-treated rats**.

Further insight into the suitability of CO-610 as a surrogate indicator of hepatic fibrosis was gained by measuring mean arterial pressure (MAP) and portal pressure (PP) in 20 of the 68 CCl_4_-treated rats included in the protocol. This subset of animals did not differ from the whole group of fibrotic/cirrhotic rats in terms of fibrosis staging (seven rats with mild/moderate fibrosis, three rats with severe fibrosis and ten rats with cirrhosis), range of CO3-610 values (37.3 ± 5.6 ng/ml, 55.1 ± 6.3 ng/ml and 72.2 ± 6 ng/ml, respectively) or correlation coefficient with hepatic collagen content (*r* = 0.71; *P* < 0.001). As previously observed, the increased disruption in the hepatic architecture was associated with a progressive deterioration in systemic hemodynamics. In fact, animals with cirrhosis had frank hypotension (MAP 96.5 ± 2.4 mmHg; *P* < 0.001) compared with control rats (MAP 124.6 ± 2.4 mmHg). Accordingly MAP inversely correlated with serum CO3-610 values in CCl_4_-treated rats (*r* = -0.59; *P* < 0.01) (Figure 4A). However, the most interesting finding of this investigation was that the serum concentration of CO3-610 depicted a higher direct relationship with the degree of portal hypertension in fibrotic/cirrhotic animals (*r* = 0.84; *P* < 0.001) (Figure 4B).

### Collagen types I and III are overexpressed in the hepatic tissue of CCl_4_-treated rats

**Figure 5:**
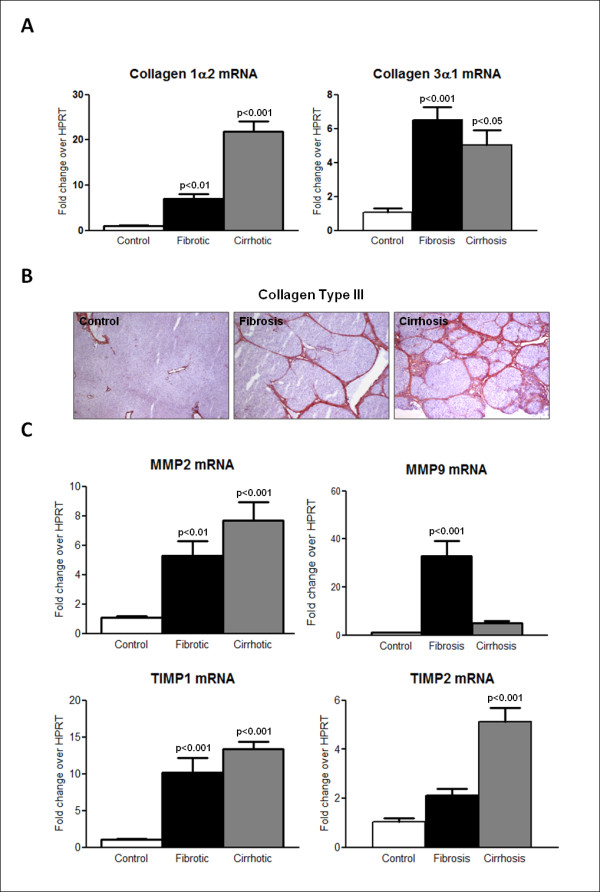
**Hepatic expression of predominant collagen types and turnover regulators of extracellular matrix (ECM) in the study rats**. Animals were grouped as control, fibrosis and cirrhosis. Rats with mild, moderate and severe fibrosis were analyzed together as the fibrosis group. **(A)** Collagens I and III transcription levels quantified by real-time PCR. **(B)** Immunolocalization of collagen type III in liver tissue. **(C)** Hepatic gene expression of matrix metalloproteinase (Mmp)-2 and Mmp9, and tissue inhibitor of matrix metalloproteinase (Timp)-1 and Timp2 by real-time PCR. Data are expressed as mean ± SEM of the group, and *P*≤0.05 was considered significant (on top of the columns). Significance is denoted versus the control group.

As expected, the expression of collagen 1α2 (Col1α2) and 3α1 (Col3α1) mRNA was generally enhanced in fibrotic/cirrhotic rats, but the pattern and degree of changes clearly differed between the two transcripts (Figure 5A). Collagen 1α2 mRNA expression increased in parallel with the worsening of the liver disease. In cirrhotic rats this enhancement was around 20 times higher than that in controls. This increase in the expression of collagen 3α1 mRNA reached its peak in the animals with fibrosis, and there was no worsening of this level in the liver of rats with cirrhosis. Because CO3-610 is a degradation product of collagen type III, we next examined the hepatic content of collagen type III in the liver of CCl_4_-treated rats. Gene activation of collagen 3α1 mRNA was associated with a progressive deposition of collagen III fibers in the hepatic tissue of animals with induced fibrosis/cirrhosis protocol (Figure 5B),

### Genes involved in ECM turnover are induced in the liver of CCl_4_-treated rats

Following hepatic injury, increased matrix deposition of collagen fibers results from an altered balance between the activity of cleavage proteases such as MMPs and their endogenous inhibitors, the TIMPs. Therefore, we sought to determine expression of the rat genes Mmp2, Mmp9, Timp1 and Timp2 in the liver of CCl_4_-treated rats with different degrees of cirrhosis (Figure 5C). Reverse transcriptase PCR analysis revealed marked Mmp2 and Mmp9 mRNA overexpression in fibrotic rats compared with control animals. A similar degree of Mmp*2* mRNA expression was apparent in cirrhotic rats; however, the abundance of the Mmp9 transcript was clearly attenuated in this group of animals. Timp1 and -2 mRNAs were also significantly overexpressed in the liver of CCl_4_-treated rats, with the higher intensity being found in cirrhotic animals.

## Discussion

It has recently been reported that human MMP-9 can perform the initial cleavage of native collagens I and III, with a clear substrate preference for type III over type I [16]. A number of specific sequences of type III collagen generated by MMP-9 have been identified previously [17]. One of these, the neoepitope CO3-610, is also produced in rodents [13]. We previously reported that CO3-610 levels are significantly increased in response to BDL in Sprague-Dawley rats [14]. Rats had serum levels 2.3 times higher than those of matched sham controls at 2 and 4 weeks after surgery. Similarly, when we investigated how CO3-610 responds to CCl_4_ intraperitoneal injection, we found that serum levels doubled after 4 weeks of treatment, and remained significantly higher than those of controls until week 8 [18].

In the present study, we found that fibrosis induced by CCl_4_ inhalation in Wistar rats also resulted in increased expression of the 25 kDa CO3-610 circulating fragment (Figure 2). The fragment could be quantified by serum ELISA, and used to differentiate the stages of hepatic fibrosis in this experimental group. In particular, the study marker could discriminate advanced fibrosis from cirrhosis, and both from mild/moderate fibrosis and normal liver conditions (Figure 3A). This suggests that CO3-610 may be of use in advanced disease diagnosis, in the prognosis of liver-related events (hepatic coma or liver-related death), or as a marker of treatment efficacy in patients with cirrhosis. In the diagnosis of advanced fibrosis, CO3-610 seems to add little extra value to the already existing serum and imaging markers; however, as a marker of treatment efficacy, it merits further research considering that currently the greatest limitation in bringing new antifibrotic drugs to the clinic is the lack of clinical trial end points [7]. In contrast to our previous studies in models of acute liver disease [14,18] in which CO3-610 levels quickly responded to the treatment in the context of high associated mortality, this study allowed us to observe how CO3-610 levels correlated with histological fibrosis along a wide range of fibrosis values (Figure 3B), and how CO3-610 can effectively discriminate stages of severe fibrosis and cirrhosis from mild/moderate or no fibrosis, and between both advanced stages of disease (Figure 3A).

A key finding in this paper is the relationship between serum CO3-610 and PP, which was the strongest of all variables studied. The assessment of hemodynamic parameters, together with liver biopsy examination, is a very important tool for staging and prognosis in liver disease. Furthermore, a recent study in patients with hepatitis C virus after liver transplantation determined that a hepatic venous pressure gradient value of 6 mm Hg or higher was accurate at identifying patients at risk of disease progression (area under the curve 0.96) [19]. To our knowledge, HA is the only serum marker that has been confirmed as an independent predictor of portal hypertension in patients with liver disease [20]. In our study, compared with HA measurements in serum, there was a stronger relationship between CO3-610 and hepatic collagen content during fibrosis, This is important because HA is an ECM component whose serum levels are closely associated with matrix deposition, and it is generally considered one of the best available liver fibrosis biomarkers. HA has been thoroughly studied in both preclinical and clinical settings, and continues to be tested in human liver disease as part of algorithmic models that include other serum markers and clinical variables [8,21,22].

Interstitial collagens type I and III are the main components of the scar matrix that replaces the basal membrane of the subendothelial space of Disse and sinusoid during liver fibrosis [23,24]. Type I collagen transcription is considered a sensitive marker of active fibrogenesis, with increased levels seen at an early stage in the process [25,26]. In the study group, Col1α2 transcription behaved as a quantitative index of fibrosis progression, whereas type III collagen transcription was activated in fibrosis, but remained at similar levels in cirrhosis (Figure 5A). This differential profile is not apparent when animals are grouped according to the time course of CCl_4_ exposure [24]. Type III collagen protein accumulation continued to increase in cirrhotic tissue (Figure 5B), probably due to decreases in interstitial collagen degradation in advanced disease. Changes in the activities of MMPs are considered one of the main factors contributing to excess collagen deposition in liver fibrosis [27]. We found a general overexpression of both gelatinases during fibrosis (Figure 5C), in agreement with previous reports [28-30]. However, rat Mmp9 gene expression was no longer induced in cirrhotic animals, but returned to control values. Similar expression profiles have been published in previous reports [31,32], with Mmp9 increases being associated with early liver injury and inflammatory reaction, but not with the stage of fibrosis, and they disappeared in advanced fibrosis. Instead, Mmp2 levels increased with tissue inflammation, and were sustained at later stages. It is thought that the initial role of MMP-9 in liver fibrosis is the degradation of the normal basolateral matrix, and that subsequent decreases in ECM breakdown by MMP-9 lead to ECM accumulation and exacerbation of liver fibrosis. CCl_4_ treatment also induced increases in rat Timp1 and -2 expression (Figure 5C). Interestingly, Timp2 increases were significant only in cirrhotic rats. Taken together, these results suggest that rat liver Mmp9 production and activity may reach their peak during fibrosis, but not cirrhosis. However, relevant Mmp9 activity also depends upon release of the latent form of the enzyme from cellular stores, and is heavily regulated by interactions with different tissue factors [33].

## Conclusions

The results presented in this paper indicate that the type III collagen neoepitope CO3-610 closely correlates with hepatic collagen content and portal pressure in rats with fibrosis. Taking into account that the CO3-610 amino acid sequence is identical in rodent and human type III collagen, our findings suggest that this peptide could ultimately be a useful non-invasive biomarker of fibrosis in patients with liver disease.

## Methods

The study was performed according to the criteria of the investigation and ethics committee of the Hospital Clinic, Barcelona, Spain.

### Induction of cirrhosis in rats and experimental procedures

The study was performed on 68 male Wistar rats with different degrees of fibrosis and 20 control Wistar rats (Charles-River, Saint Aubin les Elseuf, France). Fibrosis was induced by repetitive CCl_4_ inhalation [34]. The rats were fed *ad libitum* with standard chow and given water containing phenobarbital 0.3 g/L as drinking fluid. Animals were exposed to a CCl_4_ vapor atmosphere twice a week, starting with 0.5 minutes per exposure. The duration of exposure was increased by 1 minute after every three session until it reached 5 minutes, which was used until the end of the investigation. To examine the effects of variable degrees of hepatic fibrosis, the CCl_4_-treated rats were examined at weeks 8 (n = 13), 13 (n = 26), 16 (n = 13) and 19 (n = 16) after starting the fibrosis-induction protocol. For the analysis of results, all treated animals were grouped according to thresholds of histological fibrosis (Figure 1). Control rats were studied after similar periods of phenobarbital administration alone.

When scheduled, animals were anesthetized and a hemodynamic study was performed when indicated. A subgroup of 20 rats was selected to perform these measurements before termination to measure MAP and PP. A blood sample was then obtained from all animals to measure osmolality, electrolytes, standard parameters of liver function, and serum concentrations of HA and CO3-610. Thereafter, the animals were killed by isofluorane overdose (Forane, Abbott Laboratories S.A., Madrid, Spain), and hepatic samples were obtained from the middle liver lobe, which were immediately frozen in liquid nitrogen until further analysis of mRNA expression, or fixed in 10% buffered formalin for further analysis with hematoxylin and eosin stain, Sirius red stain and immunohistochemistry.

### Hemodynamic measurements

Rats were anesthetized with barbiturate 100 mg/kg body weight (Inactin^®^; Sigma-Aldrich Chemie Gmbh, Steinherim, Germany) and a polyvinyl catheter (PE-50; ; Becton-Dickinson, Franklin Lakes, NJ, USA) was implanted in the left femoral artery. The arterial catheter was connected to a very sensitive transducer (Hewlett Packard, Avondale, PA, USA) that was calibrated before each study. Hemodynamic parameters were allowed to equilibrate for 30 minutes, and MAP and heart rate values were recorded for two periods of 30 minutes. Each value represented the average of two measurements. A midline abdominal incision (20 mm) was made, and a PE-50 catheter was placed in the portal vein through an ileocolic vein. After verifying free blood reflux, the catheter was fixed to the mesentery with cyanoacrylate glue, and PP was measured. Animals were killed by isofluorane overdose, and tissue specimens were collected.

### Quantification of fibrosis

Liver sections 4 μm thick were stained in 0.1% Sirius red F3B (Sigma-Aldrich, St. Louis, MO, USA) in saturated picric acid (Sigma-Aldrich). The relative fibrotic area, expressed as a percentage of total liver area, was assessed by analyzing 36 fields of Sirius red-stained liver sections per animal. Each field was acquired at 10× magnification with a microscope ( E600; Nikon, Tokyo, Japan) and digital camera (RT-Slider Spot; Diagnostic Instruments, Sterling Heights, MI, USA). Results were analyzed using imaging software (ImageJ, NIH). To evaluate the relative fibrosis area, the measured collagen area was divided by the net field area and then multiplied by 100. Subtraction of the vascular luminal area from the total field area yielded the net fibrosis area [35].

### CO3-610 immunoprecipitation from serum

Serum samples from rats treated with CCl_4_ for 19 weeks and from non-treated animals were analyzed in parallel. Briefly, 1.5 μg/well of biotinylated anti-CO3-610 antibody was used to coat streptavidin 96-well plates (Thermo Scientific Pierce, Rockford, IL, USA). Serum samples (125 μL) were diluted 1:1 in immunoprecipitation buffer, added to the wells, and incubated for 1 hour at room temperature. Antibody-antigen complexes were removed from non-specific binding, and the antigen eluted from the plates according to the manufacturer's instructions. For western blotting analysis, 1.4 μl of DTT 1 mol/l was added to 35 μL of each eluted sample, and then heated for 5 minutes at 95°C. The heated samples were loaded onto 15% SDS gels, resolved, and transferred to nitrocellulose membranes using 60 mA for 90 minutes. Membranes were blocked overnight in 5% milk, and incubated for a further 2.5 hours with horseradish peroxidase-labeled anti-CO3-610 antibody (diluted 1:100). After three washes of 10 minutes each, membranes were incubated for a further 1 hour with anti-mouse IgG HRP-linked secondary antibody (diluted 1:5000) followed by another three washes of 10 minutes each. Membranes were then developed with autoradiography films (GE Healthcare, Piscataway, NJ, USA) for 30 minutes.

### CO3-610 quantification in serum

Details on the design and production of the monoclonal antibody against CO3-610 and the development of the serum CO3-610 ELISA assay have been previously published [13]. In brief, 100 μl of 2.5 ng/mL C-term biotinylated peptide NB51 (KNGETGPQGP) in PBS-Tris-borate-EDTA were used to coat streptavidin plates for 30 minutes at 20°C on a 300 rpm shaker, and then removed with washing buffer. Serum samples (20 μl) were diluted eightfold in incubation buffer (10 mmol/l: 400 mmol/l Tris:Bis-Tris buffer) and added to the plates. Next, 100 μL of CO3-610 peroxidase conjugated antibody solution (1:40,000 dilution) was added to the plates and left to incubate overnight at 4°C, on a 300 rpm shaker. The next day, plates were washed five times in washing buffer, then 100 μl of 3,3',5,5'-tetramethylbenzidine was added, and the plates incubated in darkness for 15 minutes at 20°C on a 300 rpm shaker. The reaction was stopped with the addition of 100 μL of stop solution, and plates were read on an ELISA reader at 450 nm, with 650 nm as reference.

### Messenger RNA expression of Col1α2, Col3α1, Mmp2, Mmp9, Timp1 and TIMP2

Total RNA was extracted from frozen liver specimens of control and fibrotic rats (RNeasy Kit; Qiagen, Hilden, Germany), then 1 μg of total RNA was reverse transcribed using a complementary DNA synthesis kit (High-Capacity cDNA Reverse Transcription Kit; Applied Biosystems, Foster City, CA, USA). RNA concentration was determined by spectrophotometric analysis (ND-100 Spectrophotometer, Nanodrop Technologies Inc., Wilmington, DE, USA). Total RNA (1 μg) was reverse transcribed using the same complementary DNA synthesis kit described above. Specific primers and probes used for the different genes studied were designed to include intron spanning, using the Universal Probe Library Assay Design Center through the ProbeFinder software (version 2.45; Roche Diagnostics, Indianapolis, IN, USA; https://www.roche-applied-science.com/sis/rtpcr/upl/index.jsp). The primers used are shown in Table 2. Primers for rat were designed according to GenBank sequences (NM_053356.1, NM_032085.1, NM_053819.1, NM_021989.2, NM_031054.2, NM_031055.1 and NM_012583.2, respectively) and probes were designed using ProbeFinder software as above.

**Table 2:** Primers used for PCR

Primer name		Sequence 5'→3'
Col1α2	Left	AGACCTGGCGAGAGAGGAGT
	Right	ATCCAGACCGTTGTGTCCTC
Col3α1	Left	TCCCCTGGAATCTGTGAATC
	Right	TGAGTCGAATTGGGGAGAAT
Timp1	Left	CATGGAGAGCCTCTGTGGAT
	Right	TGTGCAAATTTCCGTTCCTT
Timp2	Left	GACAAGGACATCGAATTTATCTACAC
	Right	CCATCTCCTTCCGCCTTC
Mmp2	Left	GCGCTTTTCTCGAATCCAT
	Right	GGGTATCCATCTCCATGCTC
Mmp9	Left	CCTGAAAACCTCCAACCTCA
	Right	GAGTGTAACCATAGCGGTACAGG
Hprt1	Left	GACCGGTTCTGTCATGTCG
	Right	ACCTGGTTCATCATCACTAATCAC

Real-time quantitative PCR reactions were performed in duplicate and analyzed (Light Cycler 480; Roche Diagnostics). The PCR reaction mix was 10 μl total volume, comprising 1:8 cDNA dilution, 200 nmol/l primer dilution, 100 nmol/l pre-validated nine-mer probe (Universal ProbeLibrary, Roche Diagnostics) and a master kit (FastStart Taqman Probe Master Kit; Roche Diagnostics). The hypoxanthine phosphoribosyltransferase 1 gene (Hprt1) was used as a reference gene for normalization (Table 2), and water was used as negative control.

Relative quantification was calculated using the comparative threshold (CT) cycle, which is inversely related to the abundance of mRNA transcripts in the initial sample. The mean of CT duplicate measurements was used to calculate ΔCT as the difference in CT for target and reference. The relative quantity of product was expressed as fold-induction of the target gene compared with the reference gene according to the formula 2^-ΔΔCT^, where ΔΔCT represents ΔCT values normalized with the mean ΔCT of control samples.

### Type III collagen immunohistochemical analysis

Sections of frozen liver tissue 5 μm thick were cut and fixed in acetone for 10 minutes, then dried overnight at room temperature. Slides were washed in water for 5 minutes. Anti-type III collagen ab7778 primary antibody (Abcam, Cambridge, UK) was diluted 1:50 with 1% bovine albumin, and incubated on the slides for 30 minutes at room temperature, then removed with 0.1% Triton X-100 in PBS (two washes of 5 minutes each). Slides were then incubated with secondary horseradish peroxidase Envision anti-rabbit IgG antibody (Dako, Glostrup, Denmark) for 30 minutes, and washed with 0.1% Triton X-100 (2 × 5 minutes). Red staining was produced by incubation with 3-amino-9-ethyl carbazole substrate (Vector Laboratories, Burlingame, CA, USA) for 30 minutes. Slides were counterstained with Meyers hematoxylin.

### Other measurements and statistical analysis

Serum levels of HA were quantified with an enzyme-linked binding-protein assay according to the manufacturer's instructions (Corgenix Inc, Westminster, CO, USA). Serum osmolality was determined from osmometric depression of the freezing point (Osmometer 3300; Advanced Instruments, Needham Heights, MA, USA) and Na^+^ and K^+^ concentrations by flame photometry (IL 943; Instrumentation Laboratory, Lexington, MA, USA). Serum albumin, alanine transaminase and lactate dehydrogenase were measured by a chemical analyzer (ADVIA 2400; Siemens Healthcare Diagnostics, Tarrytown, NY, USA). Quantitative data were analyzed using GraphPad Prism 5 (GraphPad Software, Inc., San Diego, CA, USA). Differences between mean values obtained from CCl_4_-treated and control groups were analyzed using one-way ANOVA with Newman-Keuls *post hoc* test, and Kruskal--Wallis test with Dunn *post hoc* tests when appropriate. Correlations between serum CO3-610 values and the rest of the variables studied were analyzed with the Pearson two-tailed test. Data was expressed as mean ± SEM, and *P*≤0.05 was considered significant.

## List Of Abbreviations

Mmp/MMP, matrix metalloproteinase (rat and human, respectively); HA, hyaluronic acid; PIIINP, human N-terminal propeptide of type III procollagen; Timp/TIMP tissue inhibitor of metalloproteinase (rat and human, respectively); LB, Liver biopsy; ECM, extracellular matrix; BDL, bile-duct ligation; MAP, mean arterial pressure; PP, portal pressure; Col1**α**2, collagen type I alpha 2 in rat; Col3**α**1, collagen type III alpha 1in rat; Hprt1, hypoxanthine-guanine phosphoribosyltransferase 1 in rat; ELISA, enzyme-linked immunosorbent assay; PBS, phosphate-buffered saline

## Competing interests

TSS, EV and NB are full-time employees at Nordic Bioscience, and MAK owns stocks at Nordic Bioscience. Nordic Bioscience is currently applying for a patent relating to the use of CO3-610 as a fibrosis biomarker. The other authors declare no competing interests.

## Authors' contributions

TSS and WJ designed and conceived the study. TSS performed the CO3-610 measurements and immunoprecipitation, VR and GFV performed HA measurements, quantitative PCR and hemodynamic measurements. MMR performed the liver-collagen quantification. NB supervised and ensured CO3-610 assay performance. SV, MMR and MAK participated in data interpretation and manuscript preparation. TSS, VR and WJ wrote the manuscript. All authors read and approved the final manuscript.
